# Headache as the initial symptom in two cases of multiple myeloma: a case report and literature review

**DOI:** 10.3389/fonc.2025.1718989

**Published:** 2026-01-12

**Authors:** Denglu Liu, Minghui Du, Xiaoyu Gao

**Affiliations:** 1 Department of Neurology, Yuhuangding Hospital Affiliated to Qingdao University, Yantai, China; 2 The Second Clinical Medical College, Binzhou Medical University, Yantai, China

**Keywords:** multiple myeloma, headache, initial symptom, plasmacytoma, hyperviscosity syndrome

## Abstract

Multiple myeloma (MM) is a hematologic malignancy characterized by clonal proliferation of plasma cells. It typically manifests with bone pain, renal failure, anemia, and hypercalcemia. However, initial presentations can be atypical, potentially leading to diagnostic delays. We present two cases of MM in which the primary presenting symptom was severe and persistent headache. In both cases, headache was the presenting symptom that ultimately led to the diagnosis. The first case was attributed to hyperviscosity syndrome, while the second resulted from an intracranial plasmacytoma with dural involvement. These cases highlight the importance of including hematological malignancies in the differential diagnosis of new, severe, or atypical headaches, particularly when accompanied by nonspecific systemic symptoms such as fatigue or weight loss. Serum protein electrophoresis (SPEP) and the free light chain (FLC) ratio are critical initial investigations for suspected cases. Early recognition of such atypical presentation can significantly reduce diagnostic delay and improve patient outcomes.

## Introduction

Multiple myeloma (MM) is a malignant clonal plasma cell disease characterized by the abnormal proliferation of plasma cells in the bone marrow, secretion of monoclonal immunoglobulins (M protein) or light chains, and associated organ damage (such as bone lesions, anemia, renal insufficiency, hypercalcemia) (1, 2). Typical initial symptoms of MM mainly include bone pain (most commonly involving the spine, ribs and pelvis), fatigue due to anemia, and lower limb edema resulting from renal insufficiency (3). Although headache is a rare initial symptom of MM (<5%), existing literature seldom systematically compares its underlying pathophysiological mechanisms or addresses the diagnostic challenges in MM patients with a low bone marrow plasma cell percentage (<10%) but typical biomarkers (4). This rarity often leads to misdiagnosis or delayed diagnosis of MM, as clinicians may initially attribute headache to more common causes such as migraine, tension-type headache or other intracranial pathologies. In this study, we report two cases of MM with headache as the initial symptom, analyze the potential mechanisms of headache in MM patients, and discuss key clinical diagnostic considerations to provide a reference for clinical practice.

## Case presentation

### Case 1

A 58-year-old male presented to the neurology outpatient clinic with persistent, gradually worsening generalized headaches over the past month. His medical history included hypertension and hyperlipidaemia. Hypertension has been well-controlled with nifedipine (120-130/80–90 mmHg) for 3 years; hyperlipidemia was managed with atorvastatin (low-density lipoprotein cholesterol: 1.92 mmol/L). He reported no history of migraine or tension-type headache and denied focal neurological deficits or bone pain. Physical examination revealed no neurological deficits.

Initial laboratory tests showed elevated levels of procalcitonin, erythrocyte sedimentation rate, and C-reactive protein. Renal function and serum calcium levels were within normal limits. Head contrast-enhanced magnetic reasonance imaging (MRI) and magnetic resonance venography (MRV) revealed no abnormalities. No significant beading of red blood cells was observed in the peripheral blood smear, and the albumin-to-globulin ratio was within normal limits. Triglyceride was 3.49 mmol/L(reference range: 0.4-1.7 mmol/L), indicating an increased risk of blood viscosity. Lumbar puncture revealed an elevated cerebrospinal fluid (CSF) protein level of 586.2 mg/L (reference range: 150–450 mg/L). Serum immunoglobulin quantification showed elevated IgG levels of 20.5 g/L (reference range: 8.6-17.4 g/L) and reduced IgA levels of 0.31 g/L (reference range: 1-4.2 g/L). Serum protein electrophoresis (SPEP) results showed elevated levels of γ-globulin and M protein, with IgGκ positive. Serum light chain κ was 5.87g/L (reference range: 1.7-3.7 g/L), light chain λ was 0.43g/L (reference range: 0.9-2.1 g/L), and free light chain κ was 107 mg/L(reference range: 6.7-22.4 mg/L). Bone marrow biopsy confirmed the diagnosis of MM, with plasma cells accounting for 13% of nucleated cells.

Following consultation with the Hematology Department, the patient was transferred to the Hematology Department for further treatment. As he was diagnosed with smouldering multiple myeloma and his headache had improved, plasma exchange or chemotherapy was not initiated. After receiving symptomatic treatment for headache and neurotrophic therapy, his condition improved, and he was discharged. The transient improvement in headache with symptomatic measures, despite the hyperviscosity-related mechanism, may be attributed to mild blood viscosity elevation and potential fluctuations in M protein levels.

### Case 2

A 62-year-old female presented with a two-year history of episodic, localised headaches behind her left eye. The pain was described as a persistent pressing sensation, accompanied by left orbital pain and occasional diplopia. Her medical history includes hypertension and a history of breast cancer surgery.

Renal function (serum creatinine: 98 µmol/L) and serum calcium were within normal limits. Non-contrast head CT revealed no abnormalities, but magnetic resonance imaging (MRI) demonstrated thickening of the left lateral rectus muscle, suggesting the presence of a localised infiltrative lesion or mass. Positron emission tomography-computed tomography (PET-CT) showed no significant skeletal lytic lesions but detected increased FDG uptake in some vertebral bodies and pelvic bones (SUVmax: 5.3). The PET-CT also indicated mildly reduced kidney volume with irregular morphology. Lumbar puncture and CSF analysis were normal. Serum immunoglobulin quantification showed elevated IgG at 34.9 g/L (reference range: 8.6-17.4 g/L), with reduced IgA and IgM levels. SPEP revealed elevated γ-globulin and M protein, with IgGκ positive. Serum free κ light chain was 650 mg/L (reference range: 6.7-22.4 mg/L), free λ light chain was 16.6 mg/L (reference range: 8.3–27 mg/L), yielding an FLC ratio (κ:λ) of 39.16. β2-MG was 4.36 mg/L (reference range: 1.09-2.53 mg/L). Cytokine testing showed interleukin-10 at 7.28 pg/ml (reference range: 0-4.91 pg/ml). Bone marrow biopsy confirmed MM, with abnormal plasma cells accounting for 11.86% of nucleated cells.

She was diagnosed with high-risk active multiple myeloma, with headaches and cranial nerve palsies attributed to the tumour’s mass effect. After consultation with the Hematology Department, she was transferred to the Hematology Department for further treatment. Following initiation of the VRd regimen (bortezomib, lenalidomide, dexamethasone), the patient experienced complete resolution of her headache and diplopia.

## Discussion

These two cases illustrate distinct pathophysiological mechanisms by which multiple MM can initially present with headache, underscoring the diagnostic challenges of atypical presentations.

In Case 1, the headache was likely a consequence of hyperviscosity syndrome. The elevated serum levels of monoclonal immunoglobulins significantly increase blood viscosity, impairing microcirculatory flow and leading to tissue hypoxia, particularly within the sensitive vasculature of the brain. The classic manifestations of hyperviscosity syndrome include neurological deficits, visual changes, and mucosal bleeding. Among these, mucosal bleeding is the most common presentation, warranting particular attention. This case highlights that even in the absence of classic CRAB features (hypercalcemia, renal failure, anemia, bone lesions), severe headache can be a critical indicator of this complication, necessitating prompt diagnosis and intervention, such as plasmapheresis. For new, persistent, and progressive headaches in middle-aged or older adults without a prior history, contrast-enhanced MRI is a more sensitive first-line imaging choice to exclude sinister etiologies, including meningeal involvement or small plasmacytomas. The updated International Myeloma Working Group (IMWG) “SLiM CRAB” criteria prioritise specific biomarkers reflecting biologically significant disease over rigid plasma cell percentage thresholds. A free light chain ratio ≥100 constitutes a high-risk biomarker in itself, with a progression risk sufficient to support a diagnosis of treatment-requiring multiple myeloma. Furthermore, the resolution of the headache with simple symptomatic therapy, despite its hyperviscosity-related mechanism, should not preclude the diagnosis of a secondary headache. The improvement was likely partial and transient, and the definitive diagnosis rested on the objective findings of M protein and an abnormal FLC ratio, which identified the underlying high-risk condition.

In contrast, the headache in Case 2 was attributed to the localized mass effect of a plasmacytoma. Plasmacytomas involving the skull base, orbit, or dura mater can cause pain through mechanisms such as periosteal stretching, direct nerve compression, or sinus obstruction. The concomitant sixth cranial nerve palsy in this patient is a classic sign of a cavernous sinus or skull base lesion. This scenario underscores the indispensable role of advanced imaging, particularly contrast-enhanced MRI, in the evaluation of focal headaches accompanied by neurological deficits. The patient’s complete resolution of headache and diplopia following systemic chemotherapy with the bortezomib, lenalidomide, dexamethasone (VRd) provides strong evidence for the causal link between the plasmacytoma and the symptoms.

Central nervous system (CNS) involvement, though rare in early-stage MM, can manifest in various forms, including solitary plasmacytomas, dural masses, or leptomeningeal disease (5, 6). As illustrated by our first case and supported by literature (7, 8), cerebrospinal fluid (CSF) analysis may reveal elevated protein levels, though findings are often non-specific. Imaging remains paramount for differential diagnosis. FDG PET/CT is highly valuable for assessing the systemic burden of the disease and guiding biopsies to the most metabolically active sites (9). Furthermore, advanced MRI is critical for distinguishing MM-related involvement from other entities, such as infectious meningitis, which may present with different enhancement patterns (10, 11).

Our experience aligns with a growing body of case reports that document unusual neurological presentations of MM. These include paroxysmal unilateral neuralgiform headache with conjunctival injection and tearing (SUNCT/SUNA) (12), dural masses mimicking other tumors (13, 14), and leptomeningeal involvement with cranial nerve palsies (15, 16). A consistent theme across these reports is the need for heightened clinical vigilance. Patients over 50 years of age presenting with new-onset, refractory, or focal headaches, especially with a history of malignancy or accompanying autonomic or neurological symptoms, warrant a comprehensive investigation.

The key clinical insight from these cases is that MM should be considered in the differential diagnosis of persistent, unexplained headache, particularly in older adults. Clues from the history (e.g., constitutional symptoms, visual changes) and physical examination (e.g., retinal changes, cranial neuropathies) should prompt investigations beyond routine neuroimaging. In addition to SPEP, the comprehensive assessment of other diagnostic tools such as FLC measurement is crucial for initiating the correct diagnostic pathway, enabling timely intervention and improving patient prognosis. For the evaluation of idiopathic or focal neuralgic headaches, contrast-enhanced MRI is the preferred imaging modality.

## Conclusions

Although headache is not a typical presenting feature of multiple myeloma, it can be the heralding symptom, primarily through mechanisms of hyperviscosity or direct bony involvement by plasmacytomas. For diffuse progressive headaches, screening for hyperviscosity should prioritise serum protein electrophoresis (SPEP) and the free light chain ratio (FLC ratio). For focal headaches with cranial nerve palsies, contrast-enhanced MRI should be prioritised to exclude plasmacytoma. Bone marrow biopsy and whole-body imaging (PET-CT) are key for diagnosis, adhering to SLiM CRAB criteria even in cases with <10% myeloma cells. The VRd regimen demonstrates marked efficacy in treating central nervous system involvement in multiple myeloma, while plasma exchange proves crucial for managing plasma viscosity-related headaches. Early identification and targeted intervention can significantly improve prognosis.

## Data Availability

The datasets presented in this article are not readily available because of ethical and privacy restrictions. Requests to access the datasets should be directed to the corresponding author. Requests to access the datasets should be directed to Xiaoyu Gao, greenboulder@163.com.
